# Non-iridescent Transmissive Structural Color Filter Featuring Highly Efficient Transmission and High Excitation Purity

**DOI:** 10.1038/srep04921

**Published:** 2014-05-12

**Authors:** Vivek Raj Shrestha, Sang-Shin Lee, Eun-Soo Kim, Duk-Yong Choi

**Affiliations:** 1 Department of Electronic Engineering, Kwangwoon University, 20 Kwangwoon-ro, Nowon-Gu, Seoul 139-701, South Korea; 2 Laser Physics Centre, Research School of Physics and Engineering, Australian National University, Canberra ACT 0200, Australia

## Abstract

Nanostructure based color filtering has been considered an attractive replacement for current colorant pigmentation in the display technologies, in view of its increased efficiencies, ease of fabrication and eco-friendliness. For such structural filtering, iridescence relevant to its angular dependency, which poses a detrimental barrier to the practical development of high performance display and sensing devices, should be mitigated. We report on a non-iridescent transmissive structural color filter, fabricated in a large area of 76.2 × 25.4 mm^2^, taking advantage of a stack of three etalon resonators in dielectric films based on a high-index cavity in amorphous silicon. The proposed filter features a high transmission above 80%, a high excitation purity of 0.93 and non-iridescence over a range of 160°, exhibiting no significant change in the center wavelength, dominant wavelength and excitation purity, which implies no change in hue and saturation of the output color. The proposed structure may find its potential applications to large-scale display and imaging sensor systems.

Nanophotonic color filters based on structural coloration have been extensively studied in recent years due to their prominent applications in diverse fields, (e.g., for use in display and imaging devices, or for colorful decoration, anti-counterfeiting, automobile, textiles, etc^1^,^2^,^3^,^4^. Such structural coloration is achieved by the generation of colors via selective transmission or reflection of visible light with the assistance of specially engineered subwavelength nanostructures. A structural color filter, without resorting to the use of pigments, is believed to be ecologically friendly, featuring outstanding long-term resistance to chemically induced discoloration^1^,^4^,^5^. In order to construct such filters, numerous approaches have been attempted, including a periodic nano-hole array in a metallic film, metal-dielectric-metal resonators, an array of metallic optical nano-antennas, nanowire arrays, guided mode resonance (GMR) based structures, photonic crystals, multilayer films, Bragg stacks, and Fabry-Perot etalons^6^,^7^,^8^,^9^,^10^,^11^,^12^,^13^,^14^,^15^,^16^,^17^,^18^,^19^,^20^. These approaches, however, are deemed to be inevitably subject to a certain level of iridescence, which indicates a dependency of the perceived color on the angle of incidence or viewing angle, posing a grave challenge to the development of high-quality color displays with a wide field of view. In order to alleviate the angular dependence of such a structural color filter, various methods have been attempted encompassing a configuration using high-index materials, bio-mimetic structures, or light funneling^3^,^21^,^22^,^23^,^24^,^25^.These approaches were primarily focused on reflective-type configurations, with little attention paid to the issue of color saturation. It is noted that a transmission-type highly-angle-tolerant structural color filter is of utmost importance and needs to be urgently developed in view of its potential applications for transmissive displays and sensors. A wide-angle transmissive structural color filter exploiting grating structures was also theoretically investigated, yet it required a complicated fabrication process due to its stringent design parameters and showed severe polarization dependency^26^.

In order to demonstrate the feasibility of the use of filter devices for display or imaging applications, the angle dependence of the hue and saturation properties of the produced color should be keenly taken into account apart from the improvement of the performance in terms of transmission or reflection efficiencies. The combination of hue and saturation is commonly known as the chrominance of a color, which is tantamount to the “sense of colorfulness”. With respect to hue, color is perceived to range from red through yellow to green and blue, or to an intermediate between any pair of two contiguous colors. Color saturation is an attribute of visual perception, signifying the degree to which color sensation differs from achromatic sensation regardless of the perceived brightness. According to a colorimetric analysis, the hue is determined by the dominant wavelength of transmitted or reflected light from an object, while color saturation can be measured quantitatively with regard to excitation purity^27^,^28^,^29^,^30^. It is immensely desirable to create a non-iridescent structural color filter enabling an angle-insensitive performance, providing a highly stable hue and saturation with high excitation purity against variations in the incidence angle, incurring a negligible optical loss. Moreover, it is preferable for the device to be prepared with a large area footprint.

Apparently, in order to improve the angular dependence of the structural color filter, such schemes as drawing upon grating-mediated resonant coupling are to be ruled out owing to their inherent sensitivity to the angle of incidence. This work is devoted to the construction of a non-iridescent transmissive structural color filter, accomplishing both high transmission and excitation purity simultaneously. The proposed device incorporates triple stacked etalon resonators in dielectric films, each of which is composed of a high-index cavity in hydrogenated amorphous silicon (a-Si:H) sandwiched with a pair of SiO_2_ films. An ultra-wide angular acceptance is recognized as one of the most salient benefits pertaining to the filter, considering that the device manifests no significant change in the center wavelength, dominant wavelength, and excitation purity, resulting in no change in hue and saturation with respect to the variation in the angle of incidence. It is also stated that the center wavelength can be selected by controlling the cavity thickness. Multiple etalons, with the corresponding cavity thickness, are vertically integrated to obtain a single highly-efficient passband in the visible spectral region, giving rise to satisfactory roll-off characteristics alongside appropriate bandwidths with a negligible sideband. Moreover, we explore color saturation, in terms of the excitation purity, and hue, with respect to the dominant wavelength, of the color output available from the proposed device. It should be noted that our device allows for both a stable hue and color saturation independent of the angle of incidence. The color filter has been designed through a rigorous coupled-wave analysis (RCWA) based method, and it has been fabricated by interleaving SiO_2_ films with a-Si:H films on a glass substrate. The filter is chiefly evaluated in the aspects of transmission, angular tolerance, polarization sensitivity, hue and color saturation. The hue and saturation are monitored in terms of the dominant wavelength and excitation purity, respectively. The proposed structural color filter exhibiting aforementioned features is predicted to be readily applicable for use in display, imaging, and sensing systems.

## Results

### Transmissive Structural Color Filter Featuring Angle Independent Spectral Responses and High Non-iridescence

**Figure 1:**
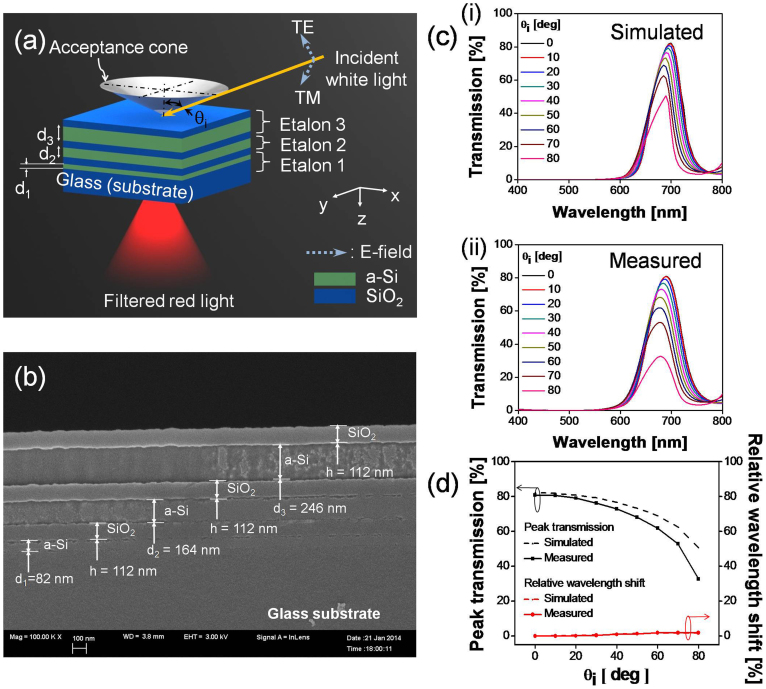
Transmissive Structural Color Filter Featuring Angle Independent Spectral Responses and High Non-iridescence. (a) Configuration of the transmissive structural color filter featuring angle-independent spectral response and high non-iridescence. (b) Scanning electron microscope (SEM) image of the fabricated filter employing three etalons based on a-Si cavity stacked one upon another interleaved with SiO_2_ layers. (c) Measured (i) and simulated (ii) spectral responses for unpolarized light at various angles of incidence θ_i_ and (d) Simulated and measured variation of peak transmission and center wavelength shift with θ_i_ for unpolarized light.

Figure 1(a) depicts the schematic configuration of the proposed transmissive color filter incorporating a multi-cavity etalon filter, which consists of three a-Si layers of unequal thicknesses interleaved with SiO_2_ layers of equal thicknesses formed on a silica glass substrate^31^. Each etalon constituting the filter is comprised of an a-Si:H cavity sandwiched between two SiO_2_ layers, serving as a partially reflecting mirror. For the light impinging upon the filter, the angle of incidence is denoted as θ_i_ while the polarization is either transverse electric (TE) or transverse magnetic (TM) in accordance with the direction of the electric field. An acceptance cone is introduced to signify the scope of the angle in which the filter exhibits no significant changes in the center wavelength, dominant wavelength, and excitation purity, thus incurring no change with respect to hue and saturation. As shown in Figure 1(a), Etalon 1 is chosen with a cavity length of d_1_ = d, while Etalons 2 and 3 adopt cavity lengths of d_2_ = 2d and d_3_ = 3d, respectively. The operation of the structural color filter is principally reliant on resonant spectral transmissions. For white light shining through the structure, constructive interference is supposed to transpire in the forward direction, giving rise to a transmission peak at wavelengths determined by the cavity length. Assuming that Etalon 1 yields a free spectral range (FSR) equivalent to Δλ, Etalons 2 and 3 accordingly provide FSRs of Δλ/2 and Δλ/3, respectively. Therefore, the device drawing upon vertically stacked triple etalons is expected to exhibit periodic transmission peaks with intervals of Δλ, where all of the three constituting elements contribute to the resonance concurrently. In this case the fundamental cavity length is determined to be d = 82 nm for a resonant wavelength at 697 nm.

In an attempt to attain a single resonant peak with extremely low angular dependence, the structure employing triple etalons was fabricated by alternately depositing three different a-Si:H films (with thicknesses of d_1_ = 82 nm, d_2_ = 164 nm, and d_3_ = 246 nm) and a 112-nm-thick SiO_2_ film on the substrate via plasma-enhanced chemical vapor deposition (PECVD). Figure 1(b) shows scanning electron microscopy (SEM) images of the fabricated device that was manufactured with high fidelity to the design. The device, involving only a few alternating layers of a-Si:H and SiO_2_, is advantageous due to its simple design, easy fabrication requiring no delicate patterning, and highly scalable dimension. Figure 1(c) (i) and (ii) present the measured and simulated transmission spectra of the color filter illuminated with unpolarized light, respectively, with the angle of incidence varying from θ_i_ = 0 to 80° in steps of 10°. The simulation was performed using an RCWA-based simulation tool. For the normal incidence, a passband located at λ_o_ = 692 nm with a peak transmission of ~80% and a 75-nm bandwidth has been practically observed, providing a satisfactorily sharp roll-off, with close correlation to the simulated case, exhibiting a pass-band centered at λ_o_ = 697 nm with an efficiency of 82% and 70-nm bandwidth. The bandwidth was observed, both in above experiments and simulations, to remain fairly unchanged throughout the range of the angle θ_i_. Figure 1(d) displays the measured and calculated peak transmissions and the relative wavelength shift with the angle θ_i_ varying from 0 to 80° in steps of 10°. The relative wavelength shift was defined as Δλ_o_/λ_o_, where λ_o_ is the center wavelength and Δλ_o_ is the shift as compared to the case with normal incidence (θ_i_ = 0°). For the entire range of angular variation, the relative wavelength shift and the degradation of the peak transmission were ~1.7% and 2.1 dB, respectively, according to the simulations, while the measured relative wavelength shift was ~1.8% and the degradation of the peak transmission was approximately 3.9 dB. The small discrepancy between the calculated and measured transmissions is attributed to the partial reflection from the glass-air interface at the bottom of the substrate, which rises sharply with an increasing angle of incidence and was not been taken into account for the simulations.

**Figure 2:**
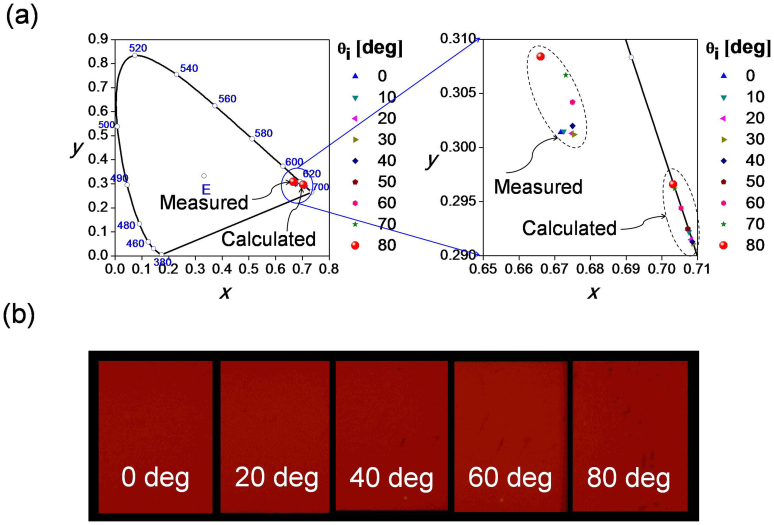
Non-iridenscence demonstrated with CIE 1931 chromaticity diagram and optical images. (a) CIE 1931 chromaticity diagram, alongside its magnified view, showing chromaticity coordinates corresponding toa filter response with respect to the angle of incidence θ_i_. (b) Optical images of the device at different angles of incidence including θ_i_ = 0°, 20°, 40°, 60°, and 80°.

In order to efficiently explore the dependency of the hue and saturation of the color output of the filter upon the angle of incidence θ_i_, as shown in Figure 2(a), the chromaticity coordinates corresponding to simulated and measured spectral responses were calculated and plotted in the standard CIE (International Commission on Illumination) 1931 chromaticity diagram^27^,^28^,^29^,^30^. The enlarged view of the diagram is also included in the figure. The dominant wavelength, which pertains to the point at which the extrapolated line joining the reference white point E (0.333, 0.333) and the chromaticity coordinates of the filter intersect the horseshoe-shaped curve, is identified first. The excitation purity is subsequently estimated as the ratio of the length of the line segment connecting the reference white point E and the chromaticity coordinates of the filter, to the length of the segment that connects the point E to the point associated with the dominant wavelength^27^,^28^,^29^,^30^. The chromaticity coordinates corresponding to the simulated and measured responses were apparently observed to suffer from no remarkable angular dependence. For the entire range of angles, the dominant wavelength and excitation purity had been calculated to be well preserved at ~627 nm and 0.99, respectively for the simulated responses, while the two parameters were observed to remain practically constant at ~623 nm and 0.93 during the experiment. It is hence guaranteed that a highly angle-insensitive performance can be acquired, exhibiting no significant degradation in hue and saturation^29^,^30^. Although peak transmission diminishes with a change in angle, the hue and saturation remain nearly constant thanks to the insignificant level of a non-resonant sideband, which will be addressed later in this paper. The small deviation may originate from unexpected fabrication errors and from the surface roughness of the films. The images of the color output for different angles of incidence, including θ_i_ = 0°, 20°, 40°, 60° and 80°, are displayed in Figure 2(b), proving there was no noticeable iridescence, as desired.

### Low Sensitivity to Input Polarization

**Figure 3:**
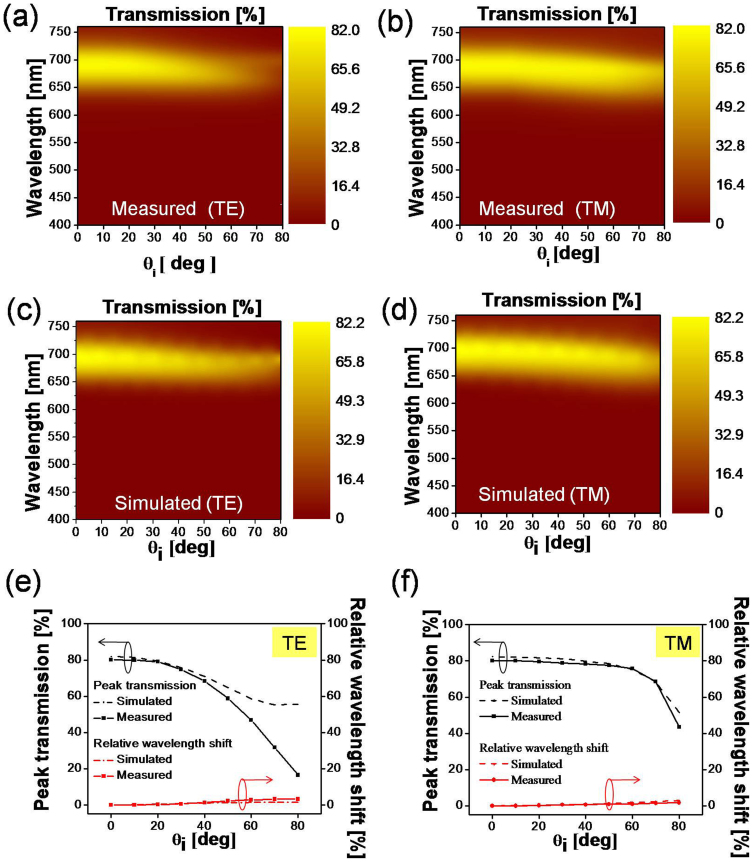
Low Sensitivity to Input Polarization. Contour map of the measured transmission in terms of the wavelength and angle of incidence for (a) TE and (b) TM polarization. Contour map of the simulated transmission with regard to the wavelength and angle of incidence for (c) TE and (d) TM polarization. Peak transmission and relative wavelength shift (Δλ_o_/λ_o_) with the angle of incidence for (e) TE polarization and (f) TM polarization.

We aim to scrutinize the influence of polarization of light upon the performance of the device as a function of the angle of incidence θ_i_. The measured contour maps for the transmission of the TE and TM polarizations for different wavelengths and angles are shown in Figure 3(a) and 3(b), respectively, while the maps for the simulations are given in Figure 3(c) and 3(d). For the angles ranging from 0° to 80°, the transfer characteristics revealed no distinct polarization sensitivity with respect to the center wavelength. Figure 3(e) and 3(f) present a plot of the variation of the peak transmission and relative wavelength shift (Δλ_o_/λ_o_) for the TE and TM polarizations, respectively. It is implied that for the case where θ_i_ = 0°, the center wavelength and peak transmission are identical for the two polarizations. The rate of decrease in peak transmission in terms of the increasing angle of incidence is severe for the TE polarization as a result of the Fresnel reflection at the air-SiO_2_ interface^32^.

### Mechanism for angle independent performance and design rule for realizing cascaded etalons

**Figure 4:**
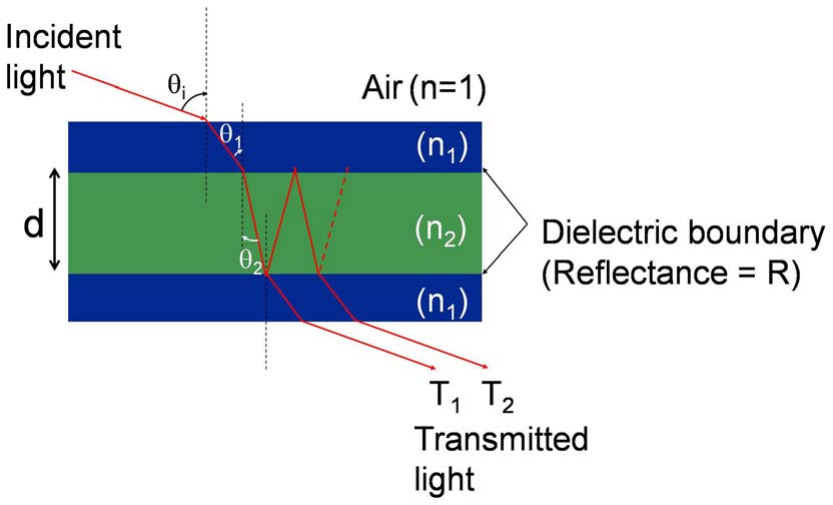
Ray-optic behavior of light in an etalon. Light approaching from the air with an angle of incidence θ_i_ undergoes successive refractions with angles of θ_1_ and θ_2_ into dielectric layers with indices n_1_ and n_2_, respectively.

The angle dependence of the proposed structural color filter was scrutinized via a simple model corresponding to a typical etalon in dielectric materials. In order to probe into the dependence of the hue and saturation upon the peak transmission, the spectral bandwidth, the location of resonant peaks and the level of non-resonant sidebands, we meticulously investigated the effect of individual parameters related to the spectral response upon the color properties in regard to the dominant wavelength and excitation purity. A typical dielectric etalon is considered, in which a dielectric cavity of refractive index n_2_ is surrounded by two dielectric layers of a refractive index n_1_ on either side, as depicted in Figure 4. Light with an angle θ_i_ emerging from the air is refracted into the dielectric layers of indices n_1_ and n_2_ at angles of θ_1_ and θ_2_, respectively. Power transmission is given by T = (1 − R^2^)/[1 + R^2^ − 2Rcosδ], where R is the reflectivity of the dielectric interface between the a-Si:H and SiO_2_ layers^32^. The phase difference between the two successive transmissions of T_1_ and T_2_ is indicated by δ = (4π/λ)n_2_dcosθ_2_ − 2*ϕ*, where *ϕ* is the phase shift imparted by the internal reflection at the interface. A transmission maximum occurs at a resonant wavelength λ_o_ for a phase difference of *δ* = 2mπ. The case where m = 1 is only considered because other resonances are sufficiently separated from it and are located in the near-infrared band. The resonant wavelength is given by 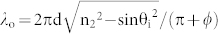. As a result, the fractional shift of the resonant wavelength with an angle *θ_i_* is found to be 
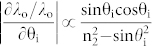
, confirming that the relative wavelength shift diminishes when the refractive index of the cavity increases.

**Figure 5:**
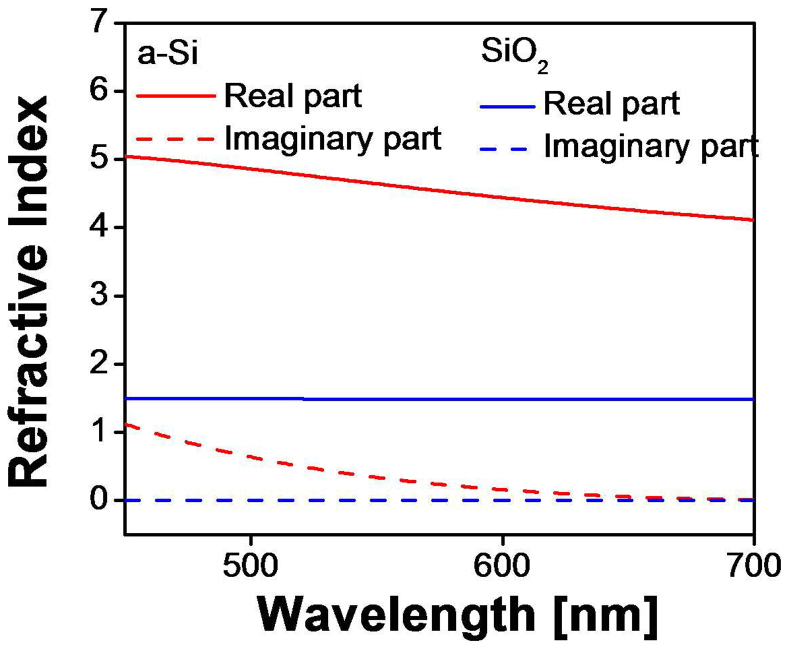
Material characteristics. Measured dispersion characteristics of a-Si:H and SiO_2_ which were used for simulation.

**Figure 6:**
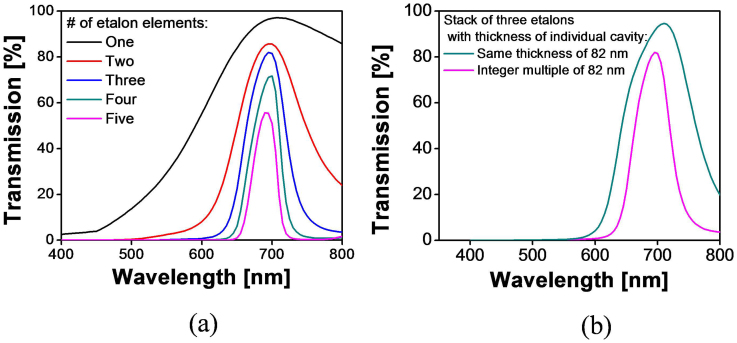
Calculated optical response for the filter with different combinations of individual dielectric etalons stacked together. (a) The calculated optical responses for different number of etalons where the cavity thicknesses of each etalon assumes integer multiples of 82 nm so as to obtain a transmission peak at λ_o_ = 697 nm. (b) The calculated optical responses corresponding to three etalons for the cases where the cavity thickness of each etalon is either kept the same or is integer multiples of 82 nm.

Based on the theoretical results given above, we resorted to the use of a high index cavity made of a-Si:H and then designed the proposed device with the help of an RCWA-based tool, thoroughly taking into account the actual dispersion for the a-Si:H cavity and SiO_2_ layer^33^. The dispersion characteristics of the a-Si:H and SiO_2_ films belonging to the device were measured in advance and were exploited for the simulations, which is shown in Figure 5. In order to make one of the multiple resonant wavelengths of the individual etalons coincide with the targeted center at ~697 nm, the cavity thicknesses of the etalons were selected to be integer multiples of 82 nm. The oxide layers enclosing the a-Si:H cavity are uniformly h = 112 nm thick. Figure 6(a) presents the theoretical transfer curves for the device with different numbers of constituent etalons, where the cavity thickness of each etalon is equivalent to integer multiples of 82 nm. For the case with three etalons, the transmission in green or blue was suppressed enough to result in a zero sideband transmission, while providing a good transmission at the resonant wavelengthof ~82%. The transmission as well as the bandwidth further reduced with an increasing number of etalons. In light of a high transmission and no significant transmission in green and blue spectral bands, we decided to use three etalons, whose cavity thicknesses were d_1_ = 82 nm, d_2_ = 164 nm, and d_3_ = 246 nm, providing an efficient passband located at ~697 nm with a satisfactorily sharp roll-off and a bandwidth of ~70 nm. It is especially noteworthy that the undesired transmission in the vicinity of the primary peak at λ_o_ = 697 nm is discovered to be substantially diminished. As presented earlier in this paper, the excellent angle tolerant filter performance was definitely attained as a result of the high-index cavities of the stacked etalons, where the relative wavelength shift was as small as 1.8%. A curiosity may arise if it is also possible to use a stack of three etalons where each of the constituent etalons has the same thickness. In order to identify the difference between the case for a cavity with the same thickness and the case for a cavity with integer multiples of a fundamental thickness, the transfer curves for the two cases were simulated and presented in Figure 6(b). The cavity thicknesses of constituent etalons were assumed to be either the same at 82 nm or integer multiples of 82 nm. The spectral response for the latter case was observed to exhibit narrower bandwidth and better suppressed sideband in the near-infrared region as compared to the former one, which is believed to be highly desirable for color rendering^21^,^34^. In view of the above results, we preferably took advantage of etalons with a cavity thickness equivalent to integer multiples of a fundamental thickness.

**Figure 7:**
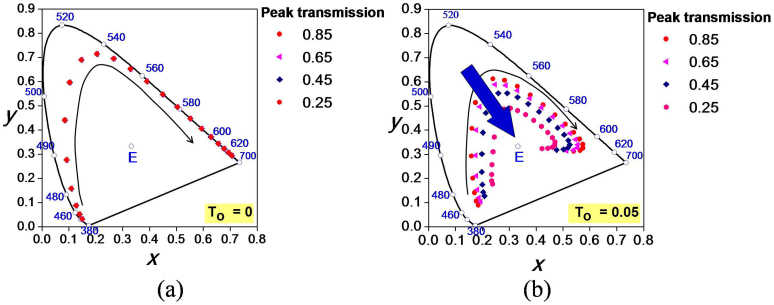
Study of origin of non-iridescent property. The variation of chromaticity coordinates in the CIE 1931 chromaticity diagram depending on the center wavelength and peak transmission, for the cases of (a) No sideband level (T_o_ = 0) and (b) Non-zero sideband level (T_o_ = 0.05) of an assumed spectral response with a normal (Gaussian) distribution defined by f(λ,λ_0_,Δω,T_0_,T_1_) = T_0_ + T_1_/exp[(λ − λ_0_)^2^(2Δω)^−2^], where λ is the wavelength, λ_o_ the center wavelength of the resonant peak, T_o_ the side band transmittance level, (T_o_ + T_1_) the peak transmission of the spectral response, and Δω the 3-dB bandwidth of the resonant peak. It is witnessed that the hue and saturation do not change if the side band transmission is maintained at zero irrespective of degradation in peak transmission.

The relationship between the spectral response of the filter and chromaticity coordinates in the CIE 1931 chromaticity diagram is investigated here, on the presumption that the wavelength shift can be minimized by utilizing a high-index cavity but the filter response experiences degradations in the peak transmission depending on the angle of incidence. We assume a normal (Gaussian) distribution for the resonance spectra as defined by f(λ,λ_0_,Δω,T_0_,T_1_) = T_0_ + T_1_/exp[(λ − λ_0_)^2^(2Δω)^−2^], where λ is the wavelength, λ_o_ the center wavelength of the resonant peak, T_o_ the side band transmittance level, (T_o_ + T_1_) the peak transmission of the spectral response, and Δω the 3-dB bandwidth of the resonant peak. The color coordinates are traced with respect to the aforementioned parameters, for an arbitrarily fixed transmittance of 85% and a bandwidth of 20 nm, in an effort to closely inspect the effect of the transfer characteristics, including the center wavelength, peak transmission and sideband level, upon the output color. Two different cases were considered for the transmission spectra in the presence and absence of the sideband level, i.e. T_o_ = 0 and T_o_ ≠ 0. In the case of a negligible sideband level, the chromaticity coordinates are identified for a peak transmission ranging from 85 to 25% in steps of 20%, with the center wavelength changing from 450 to 650 nm. As shown in Figure 7(a), the output color shifts from blue to green and eventually to red, along a locus marked with a black arrow in the clockwise direction, which closely follows the direction of the “horseshoe” contour of the color map. In Figure 7(a), it was observed that the chromaticity coordinates corresponding to the filter response with a given center wavelength remained constant regardless of the peak transmission, thus implying no change in hue and saturation in response to an alteration in the peak transmission with respect to the incidence angle. Meanwhile, as depicted in Figure 7(b), for a constant sideband level of 5% and an identical constant bandwidth of 20 nm, the chromaticity coordinate shifts with the center wavelength, sweeping over the color map from blue through green to red, in a manner similar to that of the previous case. However, as the peak transmission decreased, the coordinates related to the filter transmission with a given center wavelength deviated progressively from the initial location towards the white point (as indicated by a blue arrow), unlike in the previous case where the sideband level was zero. As a result, a change in hue and saturation was induced. For any other non-zero sideband transmissions and different bandwidths, similar behaviors were also detected, with the chromaticity coordinates deviating from the periphery of the chromaticity diagram towards the white point, resulting in a decrease in saturation. Hence, in order to achieve a constant hue and saturation irrespective of the degradation in the peak transmission resulting from the change in the incidence angle, not only should the center wavelength and bandwidth for the spectral response be fixed, but also the sideband level should be preferably maintained at zero. The non-iridescent transfer characteristics of the filter, as presented in Figure 2, can be thus explained through the fact that the device permits no noticeable transmission in the non-resonant sideband. Even though the transmission decreases with an elevating angle of incidence, as shown earlier in Figure 1, the device features no iridescence over a wide range of angles, as is intended.

## Discussion

A transmissive structural color filter that ensures non-iridescence with a stable hue and saturation over a wide angular range has been presented to provide both enhanced transmission and excitation purity. The color filter has a large footprint of 76.2 × 25.4 mm^2^ and was composed of triple stacked etalon resonators in dielectric films, each of which involves a high-index cavity in a-Si:H sandwiched between a pair of SiO_2_ films. The thickness of the a-Si:H cavity of concern is made to measure integer multiples of the fundamental length, so that all of the etalons have a common resonant wavelength. The device offered not merely an impressive transmission of ~80% at a resonant wavelength of 692 nm under the normal incidence, but also well-defined roll-off characteristics with no accompanying significant secondary peaks and substantially low polarization sensitivity. We have thoroughly examined the impact of the high-index cavity upon the broad angular tolerance of the filter, which leads to stable hue and saturation associated with the color output. For the entire range of angular variation of θ_i_ from 0° to 80°, the measured relative shift of the resonant wavelength was ~1.8%. In addition, as obtained from the colorimetric analysis, the filter delivers a drastically extended angular acceptance with a constant dominant wavelength of 623 nm in conjunction with high and stable excitation purity of 0.93 throughout the angular range of 160°, thereby indicating stable hue and saturation. One conspicuous feature of the device is that the size of the filter is highly scalable, facilitating mass production through the use of simple thin-film deposition techniques. Judging from the demonstrated performance, the device may be practically beneficial for display, imaging, and sensor applications, due to its angular insensitive characteristics that permit invariant high and stable hue regardless of variations in the incidence angle. It is worthwhile to mention that the use of a-Si:H as the high-index cavity of the etalons allowed us to demonstrate a red color, while limiting the development of blue and green structural color filters by changing the cavity thickness, since those filters are susceptible to low transmission owing to the high absorption of a-Si:H in the shorter wavelength regime. It has been however reported that, through the crystallization of deposited a-Si thin films, the extinction coefficient can be substantially diminished to improve the optical transmission in the visible band^35^,^36^. The crystallization of the deposited a-Si:H films used as the cavity or adoption of crystalline silicon for the cavity medium is potentially likely to permit non-iridescent blue and green structural color filters to offer respectable transmission. Therefore, the current approach based on a stack of etalons with high-index cavities can be safely validated. In the meantime, materials having a smaller extinction coefficient in the visible regime like TiO_2_ may be certainly regarded as an alternative to the highly absorptive a-Si material. Although it may deliver better transmission efficiency, a color filter based on stacked etalons incorporating TiO_2_ cavities is predicted to suffer from degraded angular tolerance due to its relatively low refractive index (n ~ 2.3), as compared to the case exploiting higher-index cavity such as a-Si (n ~ 3.5).

## Methods

### Simulation

Simulations of the angle resolved transmission spectra for normal and oblique incidence were performed by using rigorous coupled wave analysis (RCWA), thoroughly taking into account the complicated dispersion of a-Si:H and SiO_2_. The index of refraction of the films was checked using a reflecto-spectrometer (Filmtek4000, SCI) operating in the spectral range from 450 to 1650 nm and was incorporated into the simulations.

### Device fabrication

The proposed filter was manufactured on a glass substrate.Prior to the deposition of thin films, organic and inorganic contaminants on the substrate were cleaned successively via ultra-sonification in acetone, ethanol, and deionized water. Three a-Si:H layers, with different thicknesses of d_1_ = 82 nm, d_2_ = 164 nm, and d_3_ = 246 nm, and a 112-nm thick SiO_2_ film were alternately deposited over an area of 76.2 × 25.4 mm^2^ on the substrate via plasma-enhanced chemical vapor deposition (PECVD). PEVCD was performed using Plasmalab 100, Oxford. Here, a-Si:H was formed from silane (SiH_4_) and helium precursor gases, while silicon oxide was formed from SiH_4_, nitrous oxide (N_2_O), and nitrogen. The nominal deposition rates for the a-Si:H and SiO_2_ layers were 20 and 60 nm/min, respectively.

### Optical characterization

The cross sectional structure of the filter was observed under a high resolution scanning electron microscope (UltraPlus analytical FESEM, Zeiss), and the SEM image of the device is displayed in Figure 1(b). The thickness and index of refraction of the grown films were checked using a reflecto-spectrometer (Filmtek4000, SCI) operating in a spectral range from 450 to 1,650 nm. To examine the transmission spectra at normal and oblique incidence, a collimated light beam from a halogen lamp (Model LS-1, Ocean Optics) was irradiated upon the prepared device mounted on a motorized rotation stage, while the transmitted light was collected by a multimode fiber linked to a spectrometer (Model USB-4000-VIS-NIR, Ocean Optics). A calcite crystal polarizer (GTH 10M-A, Thorlabs) was used to polarize the incident light. All of the optical images were taken using a high definition camera (MHD-13MG5SH-D, Mintron).

## Author Contributions

V.R. performed the design, optical characterization and analysis of the device and wrote the manuscript; S.S. supervised the analysis and co-wrote the manuscript; E.S. advised and supported in preparing the manuscript; D.Y. fabricated the device and measured the thickness as well as index of refraction of the deposited films. All authors discussed the results and implications and commented on the manuscript at all stages.
